# Casting techniques of equine hand and foot synovial cavities for the development of teaching models

**DOI:** 10.3389/fvets.2025.1524549

**Published:** 2025-03-04

**Authors:** José Miguel Velásquez, Lynda Tamayo-Arango, Thamires Santos-Silva, Maria Angelica Miglino

**Affiliations:** ^1^Department of Surgery, School of Veterinary Medicine, Universidade de São Paulo, São Paulo, Brazil; ^2^Grupo de Investigación CIBAV, Escuela de Medicina Veterinaria, Facultad de Ciencias Agrarias, Universidad de Antioquia, Medellín, Colombia; ^3^Department of Veterinary Medicine, Universidade de Marília, Medellín, Brazil

**Keywords:** anatomical techniques, corrosion casting, equine arthrology, anatomical education, joint anatomy

## Abstract

Horse joints are common sites of injury, orthopedic issues, and surgical and clinical interventions. For this reason, a thorough understanding of joint anatomy, including the boundaries of their recesses and their relationships with other structures of the locomotor apparatus, is essential. This study aimed to develop cast anatomical models of the synovial structures of the equine hand and foot, compare different casting materials and visualization methods, and identify the most suitable technique for enhancing the understanding of equine limb arthrology. Additionally, an anatomical description of the synovial structures was performed to evaluate whether all relevant structures were adequately visualized using these techniques. We employed a combination of techniques, using various casting materials (methyl methacrylate, flexible epoxy resin, Smooth Cast® 300, and latex), biological maceration using dermestid beetles (Coleoptera: Dermestidae), and dissection to visualize the cavities of the carpus, tarsus, metacarpophalangeal, and interphalangeal joint. Also, the tendon sheaths of the digital flexors, extensor carpi radialis, and lateral digital flexor muscles were cast, and the podotrochlear and calcaneal subtendinous bursa were also injected. Three casting models of the metacarpophalangeal and interphalangeal joints underwent natural maceration using dermestid beetles, while the remaining joints and structures were dissected. All joints, bursa, and tendon sheaths were successfully filled with varying amounts of polymers. We concluded that joint casting is an effective and straightforward technique for producing models that enhance the understanding of the capacity and boundaries of these cavities, thereby facilitating the teaching of veterinary arthrology. We recommend methyl methacrylate as the most suitable casting material due to its ability to fill smaller cavities effectively and latex as a cost-effective option that yields good results in larger cavities. We advocate for the use of biological maceration because it avoids the use of chemicals that generate waste and toxic vapors. Future research should focus on evaluating the effectiveness of these models in enhancing the learning experience for students.

## 1 Introduction

The equine locomotor apparatus is recognized as the most frequently affected by clinical conditions (1, 2). Its primary components include bones, muscles, tendons, ligaments, and joints. Among these, joints are particularly vulnerable to disease, making clinical and surgical procedures—such as arthrocentesis, arthroscopy, and diagnostic imaging—common in equine veterinary practice. This prevalence is attributed to the large joint cavities and substantial mechanical stress experienced by joints, especially in high-performance athletic horses, where joint-related issues are a leading cause of lameness (3).

Given the clinical importance of synovial structures, a thorough and accurate understanding of joints and other synovial anatomical components is essential for equine veterinary practitioners. The anatomical study of joints (arthrology) is typically conducted through joint dissection and theoretical explanations, supported by drawings and diagrams that illustrate joint cavities. While the basic and applied anatomy of the equine limb synovial spaces is well-documented in veterinary anatomy textbooks (4, 5), undergraduate students often struggle to visualize the space occupied by the joint cavities and their surrounding structures in living animals. As a result, there is a pressing need to develop methodologies and tools that can enhance the teaching and learning of this critical subject.

The casting and corrosion technique is a widely utilized anatomical method for visualizing vessels and other hollow structures. This technique involves filling anatomical cavities with liquid materials that polymerize to form rigid negative replicas of the cavities. While casting and corrosion have been extensively applied to fill blood vessels, hollow organs, and airways (6, 7), joint casting has been less thoroughly explored. Common materials used for casting anatomical cavities include latex, silicone, resin, methyl methacrylate, and polyurethane (6).

The casting of anatomical cavities is often followed by the corrosion technique, which involves degrading the surrounding tissue to expose the rigid mold created by the casting material. Corrosion can be achieved using various maceration methods. Chemical maceration employs substances such as strong acids, bases, or enzymes (7). Physical maceration involves boiling the specimens, while biological maceration uses bacteria or the larvae of scavenging arthropods, such as dermestid beetles (8).

The primary advantages of the casting-corrosion technique include its ability to produce inexpensive, precise, and easily fabricated anatomical models (6). These models can be prepared with the help of the students themselves, allowing peer learning and enhancing students’ anatomy learning (9). The cast models improve the visualization and comprehension of typically challenging structures, and even prepare the student for the dissection of deep structures (9). However, a notable drawback of this technique is the requirement for fresh cadavers or specimens to achieve optimal distension of the anatomical structures, because cadavers or decomposed or degraded pieces, as well as previously fixed anatomical pieces, are not ideal for this type of technique because materials will not spread correctly (7).

In this study, we introduced a combination of techniques utilizing three distinct casting materials—methyl methacrylate, flexible epoxy resin, and Smooth Cast®—along with two alternative methods for visualizing joint cavities and synovial structures without relying on chemical corrosion: natural maceration using dermestid beetles and simple dissection.

This study aimed to develop cast anatomical models of the synovial structures of the equine hand and foot, compare different casting materials and visualization methods, and identify the most suitable technique for enhancing the understanding of equine limb arthrology. Additionally, an anatomical description of the synovial structures was performed to evaluate whether all relevant structures were adequately visualized using these techniques.

## 2 Materials and equipment

### 2.1 Materials

The materials used for joint injection included 5–10 mL syringes fitted with 18G needles. The casting materials employed were methyl methacrylate (Veracril®, Medellín, Colombia), flexible epoxy resin (Sumiglas, Medellín, Colombia), Smooth Cast® 300 (Smooth-On, Macungie, PA, United States), and latex (Litex T56R60, Colorquímica, Medellín, Colombia). The dyes applied were scarlet red, light blue, and bright yellow for the methyl methacrylate, flexible epoxy resin, and Smooth Cast® 300 (Sumiglas, Medellín, Colombia), respectively, and blue (Azul Novaprint SP-BR, Colorquímica, Medellín, Colombia) for the latex.

### 2.2 Animal specimens

The project was approved by the Ethics Committee on the Use of Animals of the Faculty of Veterinary Medicine at the Universidade de São Paulo (Protocol No. 9121270420). All limbs used in the study were obtained from equine specimens (*Equus ferus caballus*). The limbs, free from external injuries in the joint regions, were sourced from a local slaughterhouse and utilized immediately while fresh. The average weight of the horses from which the limbs were obtained was approximately 300 kg.

## 3 Methods

### 3.1 Joint casting process

Different limb segments were utilized in this study, as detailed below:

- **Three adult equine forelimbs**, transected at the carpus, were used to cast the digital joints: metacarpophalangeal (*metacarpophalangeae*), proximal interphalangeal (*Interphalangeae proximales*) and distal interphalangeal (*interphalangeae distales*) joints.
- **One adult equine forelimb**, transected at the middle third of the radius, was used to cast the carpal joints: antebrachiocarpal (*antebrachiocarpea*) and middle carpal (*mediocarpea*) joints, the digital flexor tendon sheath (*Vagina synovialis tendinum digitorum manus*), and the tendon sheath of the radial extensor muscle of the carpus (*Vagina tendinis musculi extensor carpi radialis*).
- **One adult equine digit**, severed from the metacarpophalangeal joint, was used to cast the podotrochlear/navicular bursa (*Bursa podotrochlearis*).
- **One adult equine hindlimb**, transected at the middle third of the tibia, was used to cast the tarsal joints: tarsocrural (*tarsocruralis*) joint, the subtendinous calcaneal bursa (*Bursa calcanea subtendinea*) and the tendon sheath of the lateral digital flexor muscle (*Vagina tendinis musculi flexoris digitalis lateralis*).

Access to the joint cavities and synovial tendon sheaths was achieved using the reference points from the arthroscopic and tenoscopic approaches described by McIlwraith et al. (10), as outlined in Table 1. The skin was first carefully dissected to directly visualize the articular capsule, bursae, and synovial sheaths. An 18G needle was then inserted into the cavity to extract synovial fluid, followed by the injection of tap water to distend the joint. The amount of water used varied depending on the size of the structure. The success of joint access was assessed by the extraction of synovial fluid and the injection of water, which distended the joints and made the joint recesses clearly visible.

**Table 1:** Reference points for joint injection.

Joint	Input	Output
Metacarpophalangeal	Dorsal pouch, abaxial to the tendon of the common digital extensor muscle.	Lateral and medial in the plantar pouches between the abaxial edge of the III metatarsal and the proximal sesamoid bones.
Proximal interphalangeal	Dorsal pouch, abaxial to the tendon of the common digital extensor muscle.	In the palmar pouch, between the abaxial edge of the distal condyles of the proximal phalanx and dorsal to the neurovascular bundle and flexor tendons.
Distal interphalangeal	Dorsal pouch, 2 centimeters proximal to the coronary border and abaxial to the tendon of the common digital extensor muscle.	In the palmar pouch, 2 centimeters proximal to the bulb of the heels, palmar and abaxial to the distal condyle of the middle phalanx and dorsal to the neurovascular bundle and the deep flexor tendon.
Antebrachiocarpal	Joint in flexed position. In the dorsal pouch, between the common digital extensor and the extensor carpal radialis tendons, midway between the distal radius and the proximal row of carpal bones.	In the dorsal pouch, 1 centimeter medial to the extensor carpi radialis tendon.
Middle carpal	Joint in flexed position. In the dorsal pouch, between the extensor carpi radialis tendon and the common digital extensor tendon, midway between the two rows of carpal bones.	In the dorsal pouch, 1 centimeter medial to the extensor carpi radialis tendon.
Tarsocrural	Joint in extended position. In the dorsomedial pouch, medial to the palpable fibularis tertius and tibialis cranialis tendons.	In the middle of the plantar pouch.
Digital flexor tendon sheath	In the proximal and lateral pouch between the palmar annular ligament and the proximal digital annular ligament, 5 mm palmar to the neurovascular bundle.	In the proximal and medial pouch between the palmar annular ligament and the proximal digital annular ligament, 5 mm palmar to the neurovascular bundle.
Calcaneal bursae	Lateral between the superficial digital flexor tendon and the long plantar ligament.	Medial between the superficial digital flexor tendon and the long plantar ligament.
Podotrochlear bursae	The flexor tendons were sectioned to expose the bursa located between the deep digital flexor tendon and the navicular bone.	Right next to the first needle.

**Figure 1:**
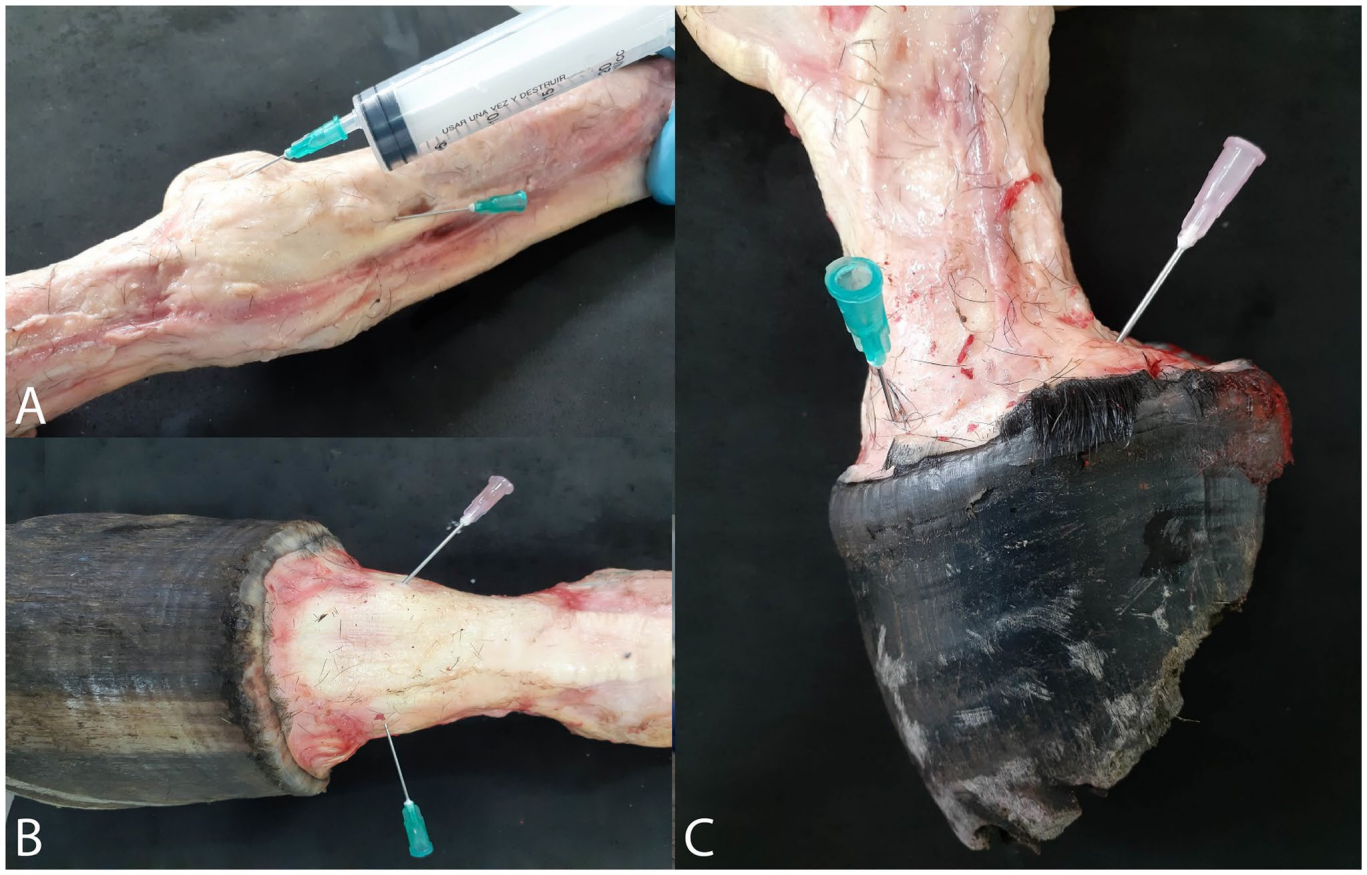
Position of the needles for injection of polymers and exit of water and air bubbles. **(A)** Metacarpophalangeal joint. **(B)** Proximal interphalangeal joint. **(C)** distal interphalangeal joint.

After the joint or synovial structure was fully distended, a second 18G needle was inserted into one or two of the articular recesses (pouches) that became visible due to the distension, as described in Table 1. This second needle was used to drain the water from the joint and create a pathway for the escape of liquids and air bubbles during the polymer injection process (Figure 1). Subsequently, all joints and synovial structures were filled to achieve maximum distension. The volume of material injected into each joint was recorded.

Different casting materials were used to fill various joints and synovial structures. The quality and success of the casting process were assessed by observing all articular recesses described in the literature following dissection or maceration. A detailed description of these recesses was provided in the results section.

#### 3.1.1 Digital joints

Three forelimbs were used for casting the digital joints (metacarpophalangeal, proximal interphalangeal, and distal interphalangeal joints), with each limb filled using a different type of casting material and dye color to facilitate differentiation and comparison of the results, as outlined below:

- Forelimb 1: Methyl methacrylate (red dye)
- Forelimb 2: Flexible epoxy resin (blue dye)
- Forelimb 3: Smooth Cast® 300 (yellow dye)

#### 3.1.2 Carpal joints

One forelimb was used for the injection of the carpal joints. Blue-stained latex was used for both the antebrachiocarpal and middle carpal joints. Additionally, the tendon sheath of the radial extensor muscle of the carpus and the digital flexor tendon sheath of the same limb were also filled with blue-stained latex.

#### 3.1.3 Podothroclear bursae

For the podotrochlear bursae, the distal part of a limb, cut at the metacarpophalangeal joint, was used. The superficial and deep digital flexor tendons were severed to allow direct visualization of the bursa, which was then filled with blue-stained latex.

#### 3.1.4 Tarsal joints

One hindlimb was used for the tarsus. Red-stained latex was injected into the tarsocrural joint and the subtendinous calcaneal bursa. Additionally, the tendon sheath of the lateral digital flexor muscle was filled with blue-stained latex.

### 3.2 Natural maceration

The three forelimbs used for casting the digital joints underwent natural maceration facilitated by dermestid beetles (Coleoptera: Dermestidae). The limbs were placed in two separate boxes, each maintained under slightly different environmental conditions, as described.

**Figure 2:**
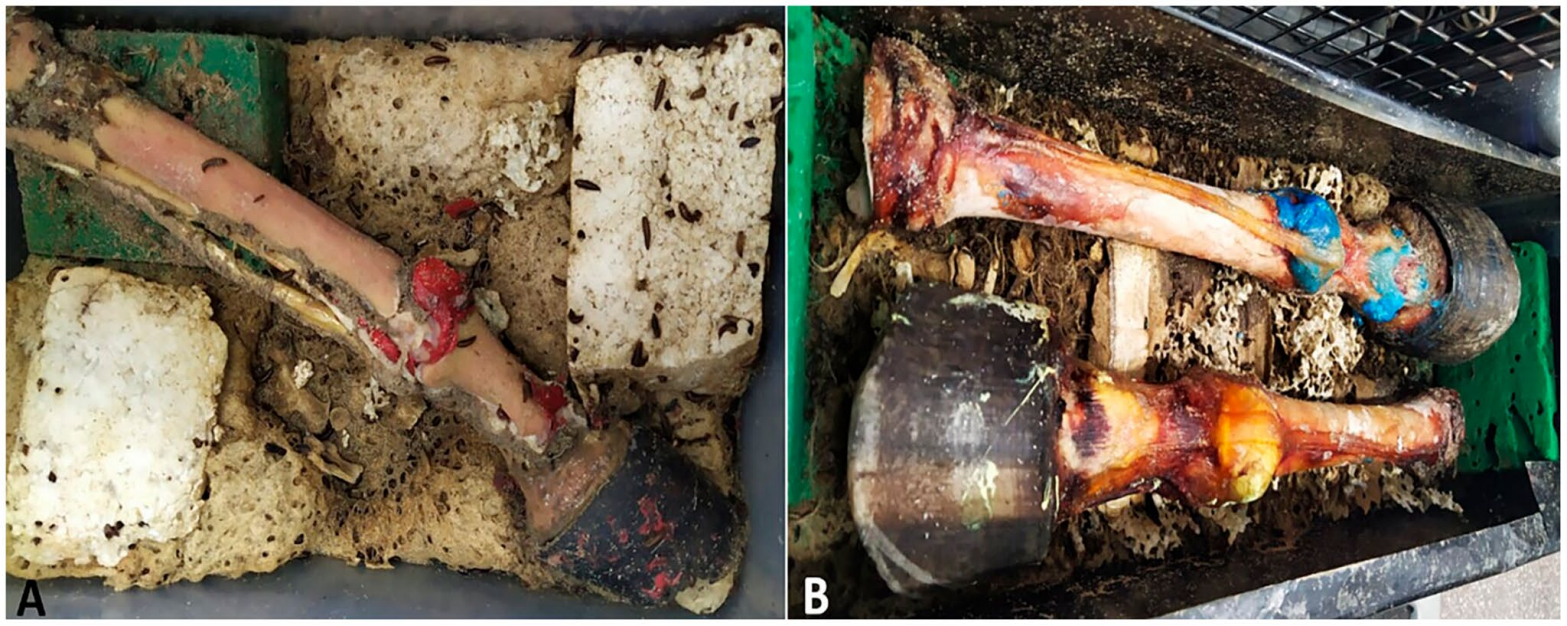
Maceration process with dermestid beetles. **(A)** Box 1 with piece filled with methyl methacrylate. **(B)** Box 2 with pieces filled with flexible epoxy resin (blues) and Smooth Cast® 300 (yellow).

In Box 1, the limb injected with methyl methacrylate was kept at a temperature of 28.8°C and a relative humidity of 45% (Figure 2A).

In Box 2, the limbs injected with flexible epoxy resin and Smooth Cast® 300 were maintained at a temperature of 29.8°C and a relative humidity of 42% (Figure 2B).

The specimens were monitored throughout the maceration process by the dermestid beetles. In Box 1, the limbs were left until all soft tissues were completely removed, while in Box 2, the limbs were monitored until most soft tissues were disintegrated, leaving some tendons and ligaments partially preserved due to desiccation. The quality and success of the maceration process were evaluated based on the extent of soft tissue breakdown, enabling clear observation of all articular recesses described in the literature.

### 3.3 Dissection

The remaining specimens were preserved by immersing them in the fixation solution described by Tamayo-Arango and Garzón-Alzate (11). After 2 weeks of immersion, the specimens were removed, and the synovial capsule and ligaments were carefully dissected to expose and visualize the filled cavities.

## 4 Results

### 4.1 Casting of the digital joints

Table 2 presents the quantities of each polymer injected into the joints. Successful access to all joints was achieved through the dorsal recesses, as evidenced by the complete filling and distension of both the joints and the palmar articular recesses. The full injection allowed for clear visualization of the various synovial anatomical structures.

**Table 2:** Quantity of polymers injected on manus joints.

Joint	Methyl methacrylate	Flexible epoxy resin	Smooth Cast® 300
Metacarpophalangeal	20 mL	18 mL	14 mL
Proximal interphalangeal	10 mL	8 mL	7 mL
Distal interphalangeal	14 mL	15 mL	12 mL

**Figure 3:**
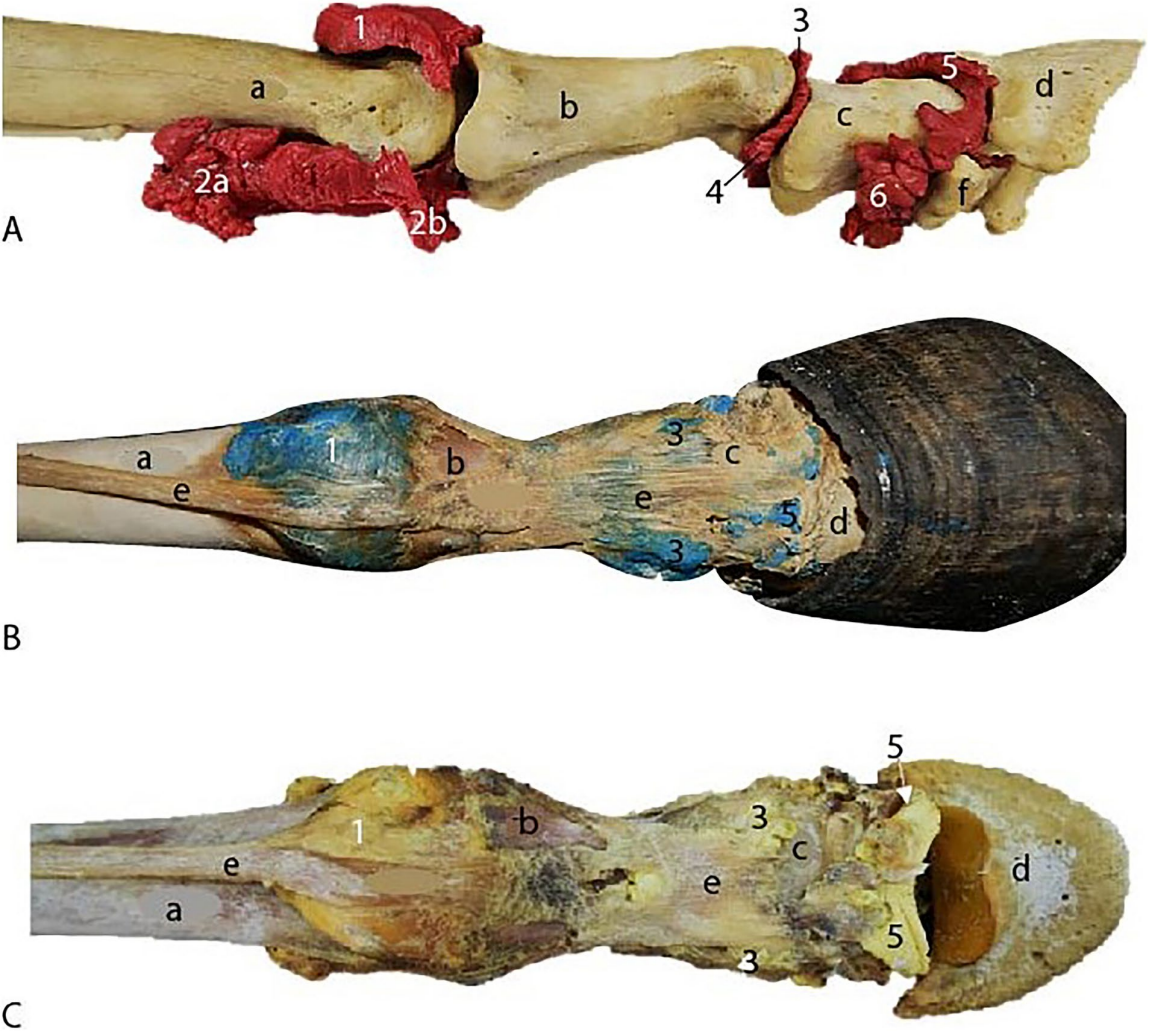
Dorsal view of the metacarpophalangeal and interphalangeal joints specimens after maceration process. **(A)** Methyl methacrylate specimen. **(B)** Epoxy resin specimen. **(C)** Smooth Cast® specimen. 1, dorsal recess of the metacarpophalangeal joint. 2a, proximal palmar recess of the metacarpophalangeal joint. 2b, distal palmar recess of the metacarpophalangeal joint. 3, dorsal recess of the proximal interphalangeal joint. 4, palmar recess of the proximal interphalangeal joint. 5, dorsal recess of the distal interphalangeal joint. 6, palmar recess of the distal interphalangeal joint. (a) III metacarpus. (b) proximal phalanx. (c) middle phalanx. (d) distal phalanx. (e), common digital extensor tendon. (f) distal sesamoid bone (navicular).

The metacarpophalangeal joint exhibited a dorsal recess that extends approximately 2 cm over the dorsal surface of the third metacarpal bone, bordered medially and laterally by the collateral ligaments. Additionally, it featured a palmar recess located between the distal end of the third metacarpal bone and the suspensory ligament, which formed a large proximal compartment and a smaller distal compartment. In all three limbs, the dorsal recesses were successfully filled, and the palmar recesses, along with their proximal and distal pouches, were distinctly delineated (Figure 3A).

The proximal interphalangeal joint was dorsally bounded by the common digital extensor tendon, laterally and medially by the collateral ligaments (Figures 3B,C), and palmarly by the straight sesamoid ligament. It showed both a dorsal and a palmar recess, with the dorsal recess extending approximately 1.5 cm over the dorsal surface of the proximal phalanx, and the palmar recess extending over the palmar and distal surfaces of the proximal phalanx. These recesses were observed in all three limbs; however, during the biological maceration process of the specimen treated with methyl methacrylate, a portion of these recesses was compromised by the dermestids.

The distal interphalangeal joint was dorsally bounded by the common digital extensor tendon, laterally and medially by the collateral ligaments (Figures 3B,C), and palmarly by the collateral sesamoid ligaments, the navicular bone, and the deep digital flexor tendon. The joint featured a small dorsal recess and a significantly larger palmar recess, which extended to the distal third of the middle phalanx. In all specimens, both the dorsal and palmar recesses were filled. In the instance of the limb filled with flexible epoxy resin, the joint was contained within the hoof. Notably, in all three limbs, the palmar recess remained adhered to the navicular bone.

### 4.2 Casting of the carpus and digital flexor tendon sheath

Table 3 displays the quantities of latex injected into each structure.

**Table 3:** Quantity of latex injected in the joints and other synovial structures.

Joint or synovial structure	Latex
Antebraquiocarpal	25 mL
Middle carpal	20 mL
Tarsocrural	60 mL
Podotroclearis bursa	10 mL
Subtendinous calcaneal bursa	15 mL
Tendon sheath of the lateral digital flexor muscle	5 mL
Tendon sheath of the radial extensor muscle of the carpus	8 mL
Digital flexor tendon sheath	42 mL

**Figure 4:**
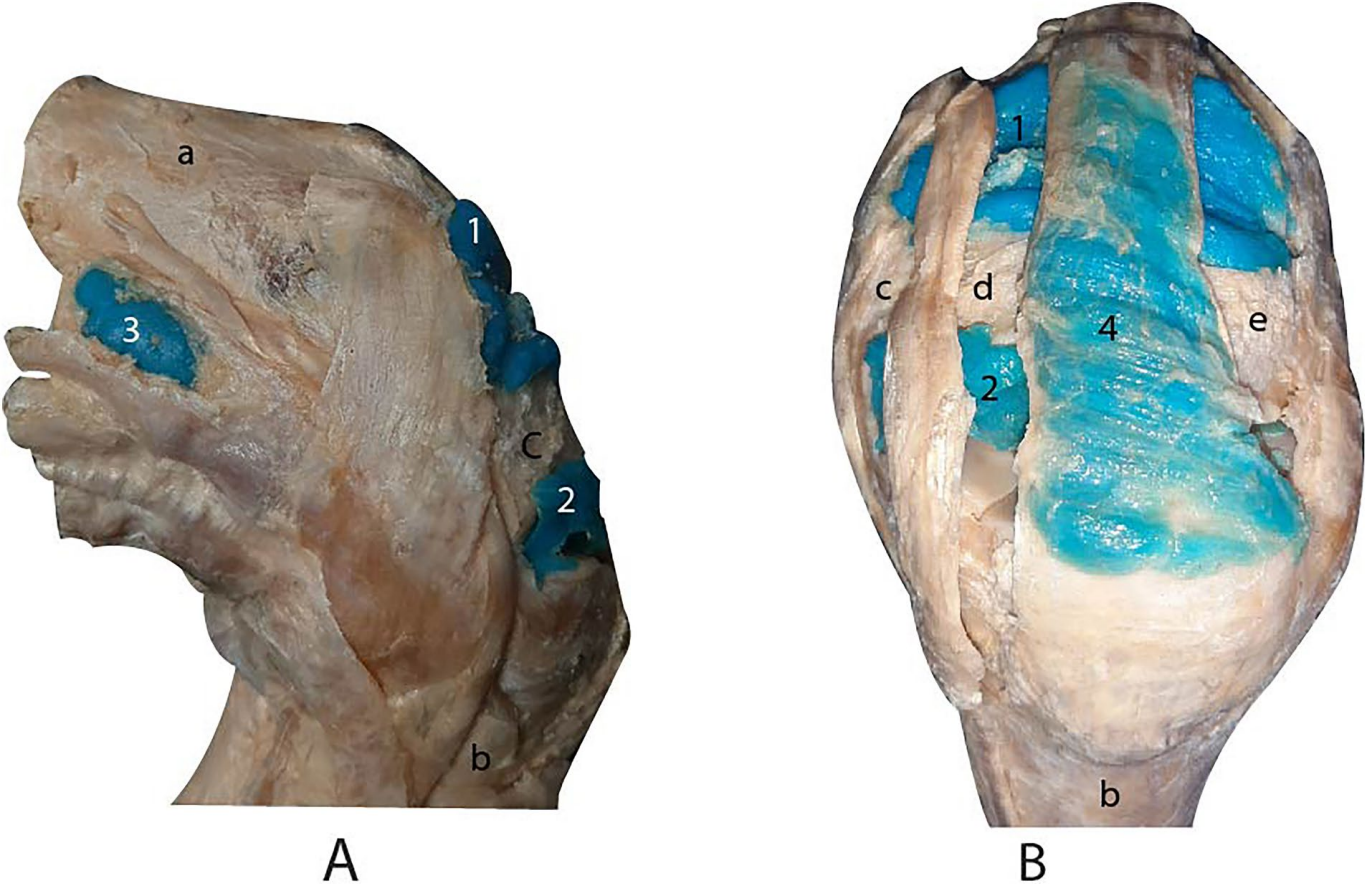
Lateral **(A)** and dorsal **(B)** views of the carpus joints after dissection. 1, dorsal recess of the antebrachiocarpal joint. 2, dorsal recess of the middle carpal joint. 3, palmar lateral recess of the antebrachiocarpal joint. 4, tendon sheath of the radial extensor muscle of the carpus. (a) radius. (b) metacarpal bone III. (c) carpal radial bone. (d) carpal intermediate bone. (e) carpal ulnar bone.

The antebrachiocarpal and middle carpal joints were filled with 25 mL and 20 mL of latex, respectively. The middle carpal joint was not completely filled due to latex diffusion occurring between the extensor retinaculum and the joint capsule. Both joints exhibited a dorsal recess and a palmar recess (Figures 4A,B). The joints were laterally bounded by the medial and lateral collateral ligaments, dorsally by the tendons of the radial extensor of the carpus, common digital extensor, and lateral digital extensor muscles, and palmarly by the joint capsule of the carpus. Additionally, the tendon sheath of the radial extensor carpi muscle was successfully filled (Figure 4B).

**Figure 5:**
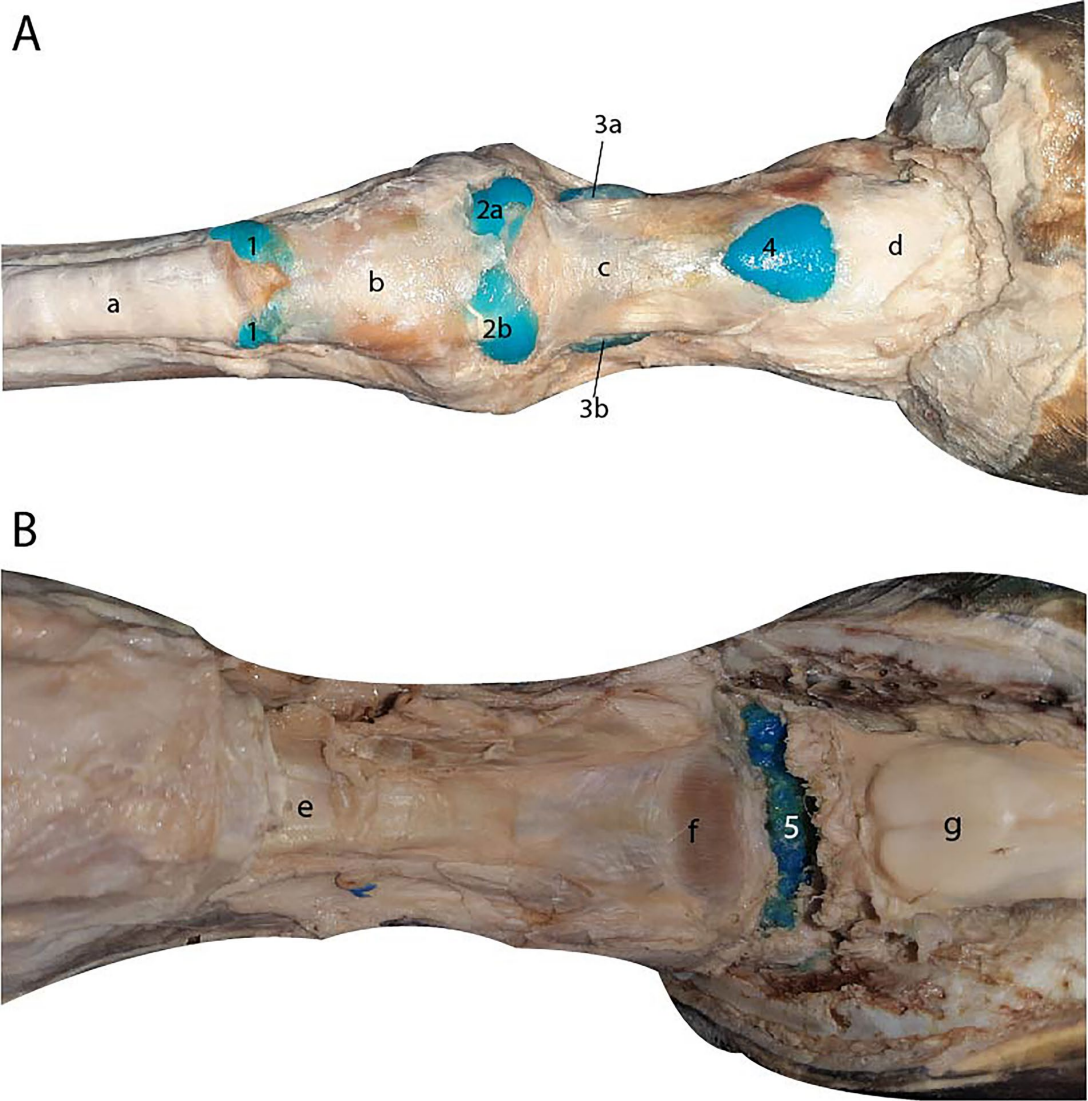
**(A)** Palmar view of the digital flexor tendon sheath. 1, proximal terminal blind recess. 2a, proximal lateral blind recess. 2b, proximal medial blind recess. 3a, distal lateral blind recess. 3b, distal medial blind recess. 4. Distal unpaired palmar recess. (a) superficial digital flexor tendon. (b) palmar annular ligament. (c) proximal digital annular ligament. (d) distal digital annular ligament. **(B)** Palmar view of the podotrochlear bursa after dissection. 5, podotrochlear bursa. (e) straight sesamoid ligament. (f) *Scutum distale*. (g) deep digital flexor tendon retracted.

The digital flexor tendon sheath was successfully infused through the recess located between the palmar annular ligament and the proximal digital annular ligament. Subsequent dissection confirmed the accuracy and efficacy of the injection, as all compartments of the sheath were distinctly visualized (Figure 5A).

### 4.3 Casting of the podothroclear bursae

Complete filling of the structure was observed following the dissection of the synovial membrane of the bursae (Figure 5B).

**Figure 6:**
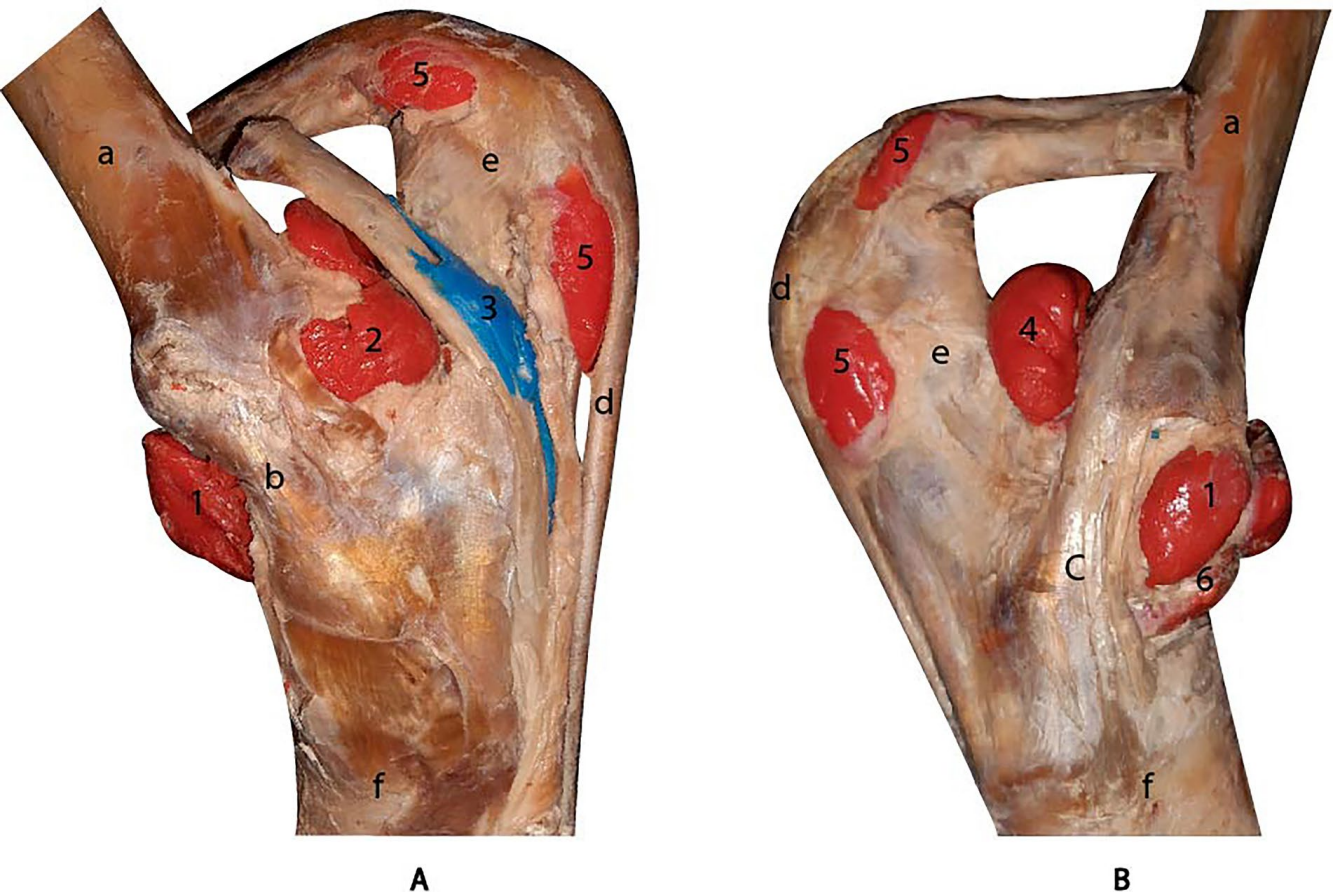
Medial **(A)** and lateral **(B)** views of the tarsus cavities after dissection. 1, dorsal recess of the tarsocrural joint. 2, medial plantar recess of the tarsocrural joint. 3, tendon sheath of the lateral digital flexor muscle. 4, lateral plantar recess of the tarsocrural joint. 5, calcaneal subtendinous bursa. 6, talocentrocalcaneal joint. (a) tibia. (b) medial collateral ligament. (c) lateral collateral ligament. (d) superficial digital flexor tendon. (e) calcaneal bone. (f) metatarsal bone III.

### 4.4 Casting of the tarsus

The tarsocrural joint was filled through the dorsomedial recess, revealing both lateral and medial plantar recesses (Figure 6). Communication with the talocalcaneocentral joint was observed, as the dorsal recess of that joint became filled during the injection of the tarsocrural joint. The joint is bounded by the medial and lateral collateral ligaments and dorsally by the tendons of the tibialis cranialis, fibularis tertius, and long digital extensor muscles. The calcaneal bursa was filled and identified between the long palmar ligament and the superficial digital flexor tendon (Figure 6). In the tendon sheath of the lateral digital flexor muscle, only a small amount of latex filled the sheath due to insufficient distension during the injection procedure.

### 4.5 Natural maceration

After 3 weeks, the three forelimbs were removed from the boxes containing dermestid beetles.

In Box 1, the specimen injected with methyl methacrylate, maintained at a lower temperature and higher humidity compared to Box 2, exhibited separated bones and well-filled joint cavities. The shapes of the dorsal and palmar recesses of all the joints were clearly visible in detail (Figure 3A).

In Box 2, the two specimens injected with flexible epoxy resin and Smooth Cast® 300 experienced desiccation of the soft tissues due to the higher temperature and lower humidity. This condition facilitated the drying and preservation of certain tendons and ligaments, including the common digital extensor tendon and the collateral ligaments. This outcome was advantageous, as it enabled detailed observation of the relationship between the joint recesses and the surrounding anatomical structures (Figures 3B,C).

### 4.6 Dissection

The dissection technique employed enabled the clear visualization of the injected cavities in the carpal and tarsal regions, as well as all associated synovial sheaths and bursae.

## 5 Discussion

Studies employing casting and corrosion techniques to examine cavities and organs are common in both human and veterinary medicine, with most focusing on vascular casting of parenchymal organs or airways. Rueda-Esteban et al. (9) utilized acrylic (methyl methacrylate), resin (epoxy and polyester), and silicone on organs such as porcine liver, as well as human and porcine lungs, achieving favorable results. Other researchers have reported the use of polyurethane to fill organs such as the lungs, hearts and kidneys of dogs, the digital veins of horses, and intestines of cats (6). Additionally, a variety of casting materials, such as epoxy resins, methyl methacrylate, latex, and silicones, have been applied in studies of animal organs including the pig liver (12), pig kidney (13), equine ocular arteries (14), rat liver (15), mouse aortic arch (16), and the equine guttural pouch (17).

Joint casting, however, has been less explored and utilized compared to other casting techniques. Nonetheless, several studies have been conducted on domestic animals. In bovines, joint casting has been employed to investigate the anatomy of the tarsocrural joint (18), carpal joints (19–21), and stifle joints (22). However, there is a relative scarcity of studies focusing on joint casting in horses.

Cheetham and Nixon (28) developed latex casts of the equine middle carpal and antebrachiocarpal joints by injecting 40 mL of latex into each joint from a 450 kg Thoroughbred gelding. In comparison, we injected 25 mL into the antebrachiocarpal joint and 20 mL into the middle carpal joint. The remarkable difference in volume may be attributed to the use of limbs from adult horses of the Colombian Criollo breed in our study, which have an average weight of 300 kg. Additionally, complete filling of the middle carpal joint was not achieved in our study, as some latex remained between the extensor retinaculum and the joint capsule. We observed the lateral and medial palmar pouches of the antebrachiocarpal joint; however, the palmar pouches of the middle carpal joint were not visible due to insufficient latex filling.

El-Bably and Abdelgalil (29) similarly produced latex casts of equine carpal joints, reporting comparable results, although they did not specify the volumes of latex injected. Alsafy et al. examined donkey carpal joints using casting and dissection methods, but they also did not provide information on the quantities used. However, their study identify communication between the middle carpal and carpometacarpal joints, consistent with findings reported by Ford et al. (38)

Regarding the casting of the tarsus joint, the only other study reporting joint casting in this region is that of Kümmerle & Kummer (39). In their study, 60 mL of silicone was injected into the tarsocrural joint of adult horses, which is consistent with the amount of latex used in our study. They also identified a plantarolateral and plantaromedial pouches within the joint, similar to our findings. Additionally, our observation of communication between the tarsocrural and talocalcaneocentral joints aligns with previous reports by various authors, explaining why a tarsocrural joint block often results in a concurrent blocking of the talocalcaneocentral joint (23).

Referring to the casting of the digital joints, several studies have described the use of latex in metatarsophalangeal joints of camels (24), metacarpophalangeal joints of sheep (25), and metacarpal/metatarsophalangeal joints of bovines (26). Latex casting has also been applied to the carpus, knee, tarsus, metatarsus and metacarpus of llamas (27).

Concerning studies on joint casting of the equine digital joints, only a limited number have been conducted, focusing either on all joints collectively or on a specific joint (28–30). However, joint casting was not typically the primary objective of these studies, and in some cases, the descriptions of the procedures results are vague and lack sufficient detail.

In our study, the palmar recess of the metacarpophalangeal joint was observed to be divided into two distinct pouches: a larger proximal pouch and a smaller distal pouch. This division was similarly noted by Amin et al. in the casting study of the donkey’s manus (31).

The smallest amount of polymer was injected into the proximal interphalangeal joint due to the limited joint cavity space, constrained by surrounding ligamentous structures (32). However, unlike our study, Radcliffe et al. (34) used 20 mL of latex to fill this joint, nearly double the amount we used. This discrepancy in the volume of polymer injected may be attributed to differences in the size and breed of the animals, as their study utilized limbs from adult purebred horses, which typically have an average weight of around 500 kg.

In the distal interphalangeal joint, we observed that the dorsal recess is broad and extends over the middle phalanx, almost reaching the proximal interphalangeal joint. This observation is consistent with the findings of Bowker et al., (40) who similarly described a wide dorsal recess when creating casts of the distal interphalangeal joint by injecting an unspecified plastic polymer through the dorsal recess, as was done in our study.

Regarding the podotrochlear bursa, this small subtendinous pocket filled with synovia fluid protects the deep digital flexor tendon from friction and pressure against the distal sesamoid (navicular) bone (33). In our study, filling the podotrochlear bursa required an adapted technique, as the standard approach described for bursoscopy could not be employed. Access to this synovial structure is more challenging due to the need for initial distension of the digital flexor tendon sheath. Furthermore, no existing literature was found describing a casting technique specifically designed for the podotrochlear bursa.

No studies were found specifically focusing on tendon sheath casting. However, these cavities are well-documented in arthroscopy literature, as they are commonly accessed for tenoscopy (9). The casting of these cavities enhances our understanding of spaces that hold clinical significance in equines.

The calcaneal bursa casting was previously conducted by Post et al., (41) who created casts from 18 limbs and reported a communication between the calcaneal bursa and the gastrocnemius calcaneal bursa in 50% of the limbs. In contrast, our study involved the casting of only one limb, and no communication between these two spaces was observed.

The techniques of repletion and maceration have been recognized as valuable for enhancing understanding of the characteristics of joint cavities. This knowledge is particularly beneficial for physical examination and diagnostic procedures, including arthroscopy (32, 34), computed tomography, and magnetic resonance imaging (31, 32, 35).

### 5.1 Comparison of maceration/visualization techniques

Chemical maceration is commonly employed following casting, utilizing agents such as potassium hydroxide (12, 16, 32), sodium hydroxide (28), sulfuric acid (6), and hydrochloric acid (13), among others. Alternatively, some authors have successfully used dermestid beetles and other necrophagous insects for biological maceration, yielding satisfactory results (8, 36, 37). The biological maceration technique employed in our study offers several advantages: it produces no chemical waste and preserves certain structures, such as ligaments and tendons, which serve as reference points for the positioning of various synovial structures. However, this method also presentes certaing challenges, as the dermestid beetles require specific care, including a habitat with controlled humidity and temperature, a continuous food supply, and proper maintenance of colony size (8).

Joint casting is an effective and straightforward technique for creating models that improve our understanding of the capacity and boundaries of joint cavities. This method facilitates the identification and comprehension of various joint recesses and their relationships with surrounding structures in the horse’s carpus, tarsus, and digital joints, as well as other important synovial structures.

The repletion and maceration/dissection technique is essential not only for anatomical education but also for research and clinical applications. It facilitates the comparison of findings with diagnostic imaging techniques and supports procedures such as anesthetic blocks for lameness diagnosis and arthroscopic surgeries.

The biological maceration technique using insects, although requiring careful maintenance of the organisms, is highly recommended as it eliminates the need for chemicals that produce waste and toxic vapors. Additionally, depending on controlled temperature and humidity conditions, this technique preserves structures such as tendons and ligaments, contributing to a more comprehensive understanding of limb anatomy in relation to synovial recesses.

For visualizing anatomical structures, we recommend using dissection to examine ligaments and biological maceration to study the capacity and distribution of synovial recesses.

### 5.2 Comparison of casting materials

Based on our experience and results, we consider methyl methacrylate to be the most suitable casting material for preparing pedagogical models of equine joints due to its superior ability to fill smaller cavities effectively, although its fragility remains a drawback compared to other materials. Conversely, latex offers a cost-effective option that provides satisfactory results when used in larger cavities. Smooth Cast® 300, while thicker in consistency and with a shorter working time due to its rapid polymerization, presents challenges for filling large cavities. In contrast, flexible epoxy resin has an intermediate density and a significant greater mechanical resistance than methyl methacrylate, resulting in more durable casts that are less prone to breakage.

All the anatomical casting models we prepared have been used repeatedly in undergraduate arthrology teaching, demostrating their value as effective pedagogical tools for enhancing the understanding of joints and other synovial structures, not only in horses but also in domestic animals in general. The cast models enable students to visualize tendon sheath, comprehend the actual spacial dimensions of joint cavities within the animal, identify joint boundaries, and explore clinical correlations, such as the communication between specific joints.

Future research should aim to evaluate the effectiveness of these models in enhancing students’ learning experiences and improving their understanding of joint anatomy and related clinical applications.

## Data Availability

The original contributions presented in the study are included in the article/supplementary material, further inquiries can be directed to the corresponding author.
